# The impact of herbal treatments on cervicovaginal human papillomavirus infection: a systematic review and meta-analysis

**DOI:** 10.1590/1806-9282.20240141

**Published:** 2024-07-19

**Authors:** Nuran Gençtürk, Aysu Yildiz Karaahmet, Dilan Cömert

**Affiliations:** 1 Istanbul University-Cerrahpaşa, Faculty of Health Sciences, Department of Midwifery – İstanbul, Turkey.; 2 Halic University, Faculty of Health Sciences, Department of Midwifery – İstanbul, Turkey.; 3 Istanbul University-Cerrahpaşa, Graduate Education Institute, Department of Midwifery, Doctoral Program – İstanbul, Turkey.

**Keywords:** HPV infection, Cervicovaginal lesions, Herbal, Treatment, Randomized controlled trial

## Abstract

**OBJECTIVE:**

This systematic review and meta-analysis aimed to investigate the effects of herbal treatments on cervicovaginal human papillomavirus infection.

**METHODS:**

A comprehensive literature search was conducted in PubMed, Scopus, Science Direct, and the Cochrane Library until December 2023, following Cochrane guidelines. Data were analyzed using the Review Manager computer program (Version 5.4.1).

**RESULTS:**

Five randomized controlled trials involving a total sample size of 662 women were included in the study. The pooled odds ratio for individuals testing negative for human papillomavirus after herbal intervention among human papillomavirus-positive patients was 1.86 (95% confidence interval (CI) 0.64–5.43), according to the fixed-effects model. Three out of the five studies indicated a significant relationship. The relationship between positive human papillomavirus infection and herbal treatments, measured by the fixed-effects model, resulted in a pooled odds ratio of 0.41 (95%CI 0.17–1.01), reporting a significant association (p=0.05). Subgroup analysis revealed a significant reduction in the relationship between herbal treatment and atypical squamous cells of undetermined significance (OR 0.16, 95%CI 0.03–0.88, p=0.04) but no significant impact on the relationship between herbal treatment and low-grade squamous intraepithelial lesion (OR 0.33, 95%CI 0.01–8.77, p=0.51).

**CONCLUSION:**

The meta-analysis suggests that herbal treatments reduce human papillomavirus infections. While herbal treatments show a significant reduction in atypical squamous cells of undetermined significance, they do not significantly impact the regression of low-grade squamous intraepithelial lesions.

## INTRODUCTION

According to the 2020 cancer statistics from the Global Cancer Observatory (GLOBOCAN) by the International Agency for Research on Cancer (IARC), cervical cancer is the fourth most common cancer globally^1^. Human papillomavirus (HPV) is the most prevalent sexually transmitted infection and forms the foundation for the development of cervical cancer neoplasia. HPV is detected in 99.7% of cervical cancer cases^2^. A large portion of HPV cases resolves spontaneously without any symptoms^2,3^. The World Health Organization (WHO) estimates the global prevalence of HPV to be between 9 and 13% of the population^3,4^. While prophylactic vaccines effectively prevent new infections, they do not eliminate existing infections^5^. HPV infections can be treated with methods such as laser, conization, and surgery. Invasive treatments are generally effective in high-grade intraepithelial lesions (HSIL) cases. However, these invasive treatments may have various side effects, such as early delivery, late miscarriages, cervical stenosis, and the possibility of recurrence^6^. Therefore, there is a need for non-invasive treatments that can eliminate lesions and HPV infections^7^. As one of these non-invasive methods, herbal treatments are reported to be effective in clearing HPV and reducing abnormal cytology associated with HPV^8,9^.

This systematic review and meta-analysis aimed to examine the impact of herbal treatments on cervicovaginal HPV infection. This study represents the first meta-analysis solely evaluating the effectiveness of herbal treatments. The meta-analysis of randomized controlled trials (RCTs) on herbal treatments for patients with cervicovaginal HPV infection was conducted to obtain more concrete results.

## METHODS

A systematic review and meta-analysis were conducted to evaluate the impact of herbal treatments on cervicovaginal HPV infection. The study aimed to address the following questions:

1. What is the effect of herbal treatments on the treatment of HPV infection?
2. What is the impact of herbal treatments on cytological regression?

### Protocol registration

The PRISMA (preferred reporting items for systematic reviews and meta-analysis statement) guidelines was followed in preparing this systematic review and meta-analysis. Throughout the study, there were no instances requiring deviation from the protocol, and the study was concluded following the protocol registered in the PROSPERO database (CRD42023491610).

### Eligibility criteria

The selection of studies adhered to the following criteria (PICOS): Participant (P): Women with cervicovaginal HPV infection. Intervention (I): Herbal interventions, including (1) oral polyphenon E; (2) curcumin vaginal capsule; (3) vaginal spray containing olive oil; (4) myrtle vaginal suppository; (5) oral epigallocatechin gallate (EGCG); and (6) their combinations. Comparison (C): Comparison with a placebo or a group not receiving any treatment. Outcomes (O): (1) Results related to the effectiveness of herbal treatments on the treatment of HPV infection and (2) results related to the effectiveness of herbal treatments on cytological regression. Study Design (S): Experimental randomized controlled studies published in English and Turkish until December 2023 were included.

Exclusion criteria encompassed individuals under 18 years old, pregnant and breastfeeding women, those with known or suspected cervical cancer, HIV-positive patients, individuals with known allergies to herbal methods, animal studies, studies with unavailable full texts, and all studies not related to herbal treatments and incorporated theoretical studies, editorial comments, non-experimental studies such as only protocol studies and review papers, and articles using measurement tools with questionable validity. The inclusion of studies was limited to those with results or outcome tables available in English or Turkish.

### Search strategy

For this systematic review, the literature search was conducted until December 20, 2023, using databases such as PubMed, Scopus, Science Direct, and the Cochrane Library.

The keywords included "HPV infection" OR "human papillomavirus" OR "cervicovaginal lesions" AND "curcumin" OR "polyphenon E" OR "myrtle" OR "epigallocatechin gallate" OR "herbal treatment." Additionally, systematic reference lists of articles and previous systematic reviews were searched.

### Selection of studies and data extraction

Data were extracted by one reviewer (DC) using a data extraction form and checked by a second reviewer (AYK). Discrepancies between the two were resolved by a third researcher (NG). General characteristics of the studies (e.g., author, country, publication year, and study design), average age of participants, sample size of groups, type of intervention, duration of follow-up, intervention method, and primary outcome variables were included for each study (Table 1).

**Table 1:** Features of the included studies.

References, country	Study design	Study period	Age	Sample size	Interventıon type	Intervention time and method	Control group	Outcomes
Aragona et al.^1^, Italy	RCT	June 2022 to August 2022	Intervention: 37.35±2.60 Control: 37.65±2.48	Intervention: 20 Control: 20	Pervistop^®^ [200 mg epigallocatechin gallate (EGCG), 400 μg folic acid (FA), 1 mg vitamin B12, and 50 mg hyaluronic acid (HA)]	Time: 3 months Method: Oral Pervistop^®^ (200 mg epigallocatechin gallate) once daily	Control	HPV clearance: Intervention group: 17/20; Control group: 5/20 Cytological regression Intervention group: 3/20; Control group: 15/20
Nikakhtar et al.^7^, Iran	RCT	November 2016 to December 2017	Intervention: 31.12±7.92 Placebo: 33.58±6.58	Intervention: 27 Placebo: 25	Myrtle	Time: 3 months Method: 20 vaginal suppositories for each month, 60 vaginal suppositories in total	Placebo	HPV clearance: Intervention group: 25/27; Placebo group: 17/25
Baleka Mutombo et al.^6^, Congo	RCT	July 2015 to July 2017	Intervention: 41.6±10.6 Placebo: 42.4±11.1	Intervention: 168 Placebo: 159	Antiviral AV2^®^	Time: 6 months Method: spraying spray on cervix	Placebo	HPV clearance: Intervention group: 73/168; Placebo group: 61/159 Cytological regression Intervention group: 5/168; Placebo group: 13/159
Garcia et al.^17^, United States of America	RCT	N/A	Intervention: 28.48±8.78 Placebo: 28.27±8.05	Intervention: 41 Placebo: 41	Polyphenon E	Time: 4 months Method: oral once daily, Polifenon E 800 mg	Placebo	HPV clearance: Intervention group: 10/41; Placebo group: 12/41
Basu et al.^18^, India	RCT	N/A	Intervention: 37.5 (35.8–39.2) Placebo: 38.3 (35.7–38.9)	Intervention: 79 Placebo: 82	Curcumin	Time: 4 months Method: Curcumin vaginal capsule (500 mg per application)	Placebo	HPV temizleme: Intervention group: 75/79; Placebo group: 81/82

RCT: randomized control trials; N/A: data not reported.

### Quality assessment

Bias risk was assessed for seven domains: random sequence generation, allocation concealment, blinding of participants and personnel, blinding of outcome assessment, incomplete outcome data, selective reporting, and other biases. Other bias sources of this study included fundamental group imbalances and potential confounding factors. The risk of bias for each study was assessed as low, high, or unclear. The bias assessment was independently conducted by two researchers (AYK and DC), and in case of disagreement, the researchers reviewed the full text together to reach a consensus.

### Data analysis

The relationships between herbal treatments and HPV parameters were estimated using pooled odds ratios (OR) and 95% confidence intervals (CI) with the Mantel-Haenszel method. Heterogeneity between studies was assessed using Cochran's Q test and Higgins' I², where I² greater than 50% indicated significant heterogeneity. Random-effects results were considered when I² was greater than 50%, and fixed-effects results were considered when I² was less than or equal to 50%. Various sensitivity analyses were conducted to assess the robustness of our results, excluding some articles considered of low quality. All analyses were performed using Review Manager 5.4.1 (The Nordic Cochrane Center, Copenhagen, Denmark). All tests were two-tailed, and a p-value less than 0.05 was considered statistically significant.

## RESULTS

### Study selection

**Figure 1:**
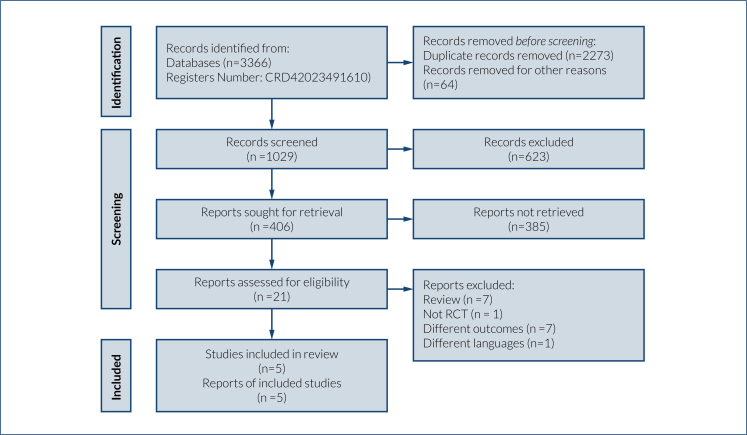
Preferred reporting items for systematic reviews and meta-analysis 2020 flow diagram for new systematic reviews which included searches of databases and registers only.

Figure 1 presents the PRISMA flowchart summarizing the literature search and study selection process. The remaining 21 full-text articles were assessed for eligibility, and five articles that met the criteria for RCTs were included in the analysis (Figure 1).

### Study characteristics

This systematic review and meta-analysis encompass five studies involving a total of 662 women, aiming to examine the impact of herbal treatments on cervicovaginal HPV infection. The studies were conducted in India^10^, the United States^11^, Iran^7^, Congo^12^, and Italy^13^. The study design for all included studies was RCTs. Table 1 provides a summary of the characteristics of the studies. In all studies within the scope of this review, herbal treatments were applied to women in the intervention group. The efficacy of herbal treatments on cervicovaginal HPV infection was assessed in all studies. In the control group, Aragona et al.^1^ did not administer any intervention, while the other four studies^7,10-12^ implemented a placebo intervention.

### Quality of bias assessment

Except for one study^13^, all studies established a sufficient method for randomly assigning participants to cognitive-behavioral intervention groups. Therefore, we assessed the risk of bias as low in this domain for these studies. Except for Aragona et al.,^1^ all studies reported sufficient allocation concealment for randomization and were evaluated as having a low risk of bias, except for one study^11^ where dropouts were balanced between intervention and control groups or were considered to have a low risk of bias due to the small number of dropouts that would not significantly impact the study. In three studies included in the meta-analysis^10-12^, we considered a high risk of bias because they discussed significant results, including adverse outcomes, and matched the reports in their records. For each included study, we explained significant concerns about other potential sources of bias not addressed in the above categories. Specifically, we sought a conflict of interest statement and a funding source. None of the studies reported any other bias risk.

### Meta-analysis

**Figure 2:**
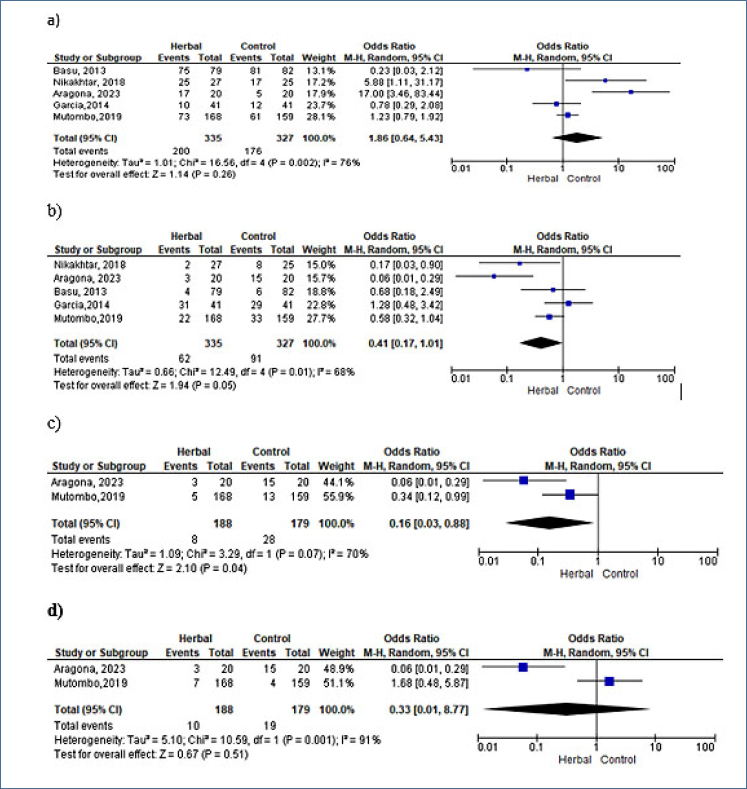
Forest plots showing the association between herbal treatment and (a) negative human papillomavirus, (b) positive human papillomavirus, (c) Atypical squamous cells rate, and (d) Low grade squamous intraepithelial lesions rate.

The meta-analysis results depicting the relationship between herbal interventions and HPV infection in women were presented as a forest plot. All included studies reported an association between herbal interventions and testing negative for HPV after the application to individuals initially testing positive. The measurements of the relationship between HPV and herbal treatments in the selected studies varied between 0.23 (95%CI 0.03–2.12) and 17.00 (95%CI 3.46–83.44). These studies showed a significant degree of heterogeneity (I2:76, p=0.002). According to the fixed-effects model, the pooled OR was 1.86 (95%CI 0.64, 5.43). Among the five studies, three demonstrated a significant relationship between herbal treatment and HPV infection (Figure 2a). All included studies reported an association between herbal interventions and a positive assessment for HPV infections. Measurements of the relationship between HPV and herbal treatments in the selected studies varied between 0.06 (95%CI 0.01, 0.29) and 1.28 (95%CI 0.48, 3.42). These studies exhibited a significant degree of heterogeneity (I2:68, p=0.01). According to the fixed-effects model, the pooled OR was 0.49 (95%CI 0.16–1.45). Among the five studies, three demonstrated a significant relationship between herbal treatment and HPV infection (Figure 2b). Two included studies reported an association between herbal interventions and atypical squamous cells (ASCUS). Measurements of the relationship between herbal treatments and ASCUS in the selected studies varied between 0.06 (95%CI 0.01–0.29) and 0.34 (95%CI 0.12–0.99). These studies showed a significant degree of heterogeneity (I2:70, p=0.07). According to the fixed-effects model, the pooled OR was 0.16 (95%CI 0.03, 0.88). Two studies demonstrated a significant relationship between herbal treatment and ASCUS (p=0.04) (Figure 2c). One included study reported an association between herbal interventions and low-grade squamous intraepithelial lesions (LSIL). Measurements of the relationship between herbal treatments and LSIL in the selected studies varied between 0.06 (95%CI 0.01–0.29) and 1.68 (95%CI 0.48–5.87). These studies exhibited a significant degree of heterogeneity (I2:91, p=0.001). According to the fixed-effects model, the pooled OR was 0.33 (95%CI 0.01, 8.77). It indicated no significant difference between herbal treatments and LSIL (p=0.51) (Figure 2d).

## DISCUSSION

This systematic review and meta-analysis delved into the efficacy of herbal treatments on cervicovaginal HPV infection. According to the meta-analysis results, herbal treatments were found to be effective in treating HPV infection. Furthermore, secondary outcomes indicated the effectiveness of herbal treatments in terms of ASCUS. However, they did not show a significant effect in regressing lesions concerning LSIL.

While HPV infection can be treated with invasive methods^14^, there is a substantial need for acceptable, safe, cost-effective, and non-surgical treatments to prevent cervical cancer. Herbal treatments emerge as one of these non-invasive methods^15,16^. In this study, herbal treatments were determined to be effective in clearing HPV infection (p=0.05). Although there is no meta-analysis specifically evaluating the effectiveness of herbal treatments in HPV treatment in the literature, a meta-analysis found a significant impact of biological and herbal studies on HPV clearance in a subgroup analysis similar to this study (p=0.01)^11^.

A literature search identified meta-analysis studies evaluating the efficacy of non-surgical treatments on HPV. The results, similar to this study, indicated a significant effect on clearing HPV: Xiong et al.^10^ (p<0.00001), Huang et al.^5^ (p <0.0001), and Zhuang and Yang^11^ (p<0.01). While these studies are up-to-date, providing a comprehensive assessment of non-surgical treatment methods, this study is crucial for focusing exclusively on the impact of herbal treatments and obtaining more specific data.

This study evaluated the regression of ASCUS and LSIL with herbal treatment methods as a secondary outcome. A regression in lesions concerning ASCUS was determined with herbal treatments (p=0.04). However, a significant effect concerning LSIL (p=0.51) could not be determined. In their subgroup analysis of a meta-analysis, Xiong et al.^5^ found a significant impact of biological and herbal studies on cytological regression similar to this study (p=0.02). The same study found the overall effectiveness of non-surgical treatments on cytological regression to be significant (p=0.001). In a meta-analysis^15^, it was determined that non-surgical treatment methods had a significantly higher level of regression in mild abnormal cytology compared with the control group (p<0.00001). The inability to obtain significant results regarding LSIL in this study may be due to the limited number of studies considered for evaluation.

### Strengths and limitations

One of the robust aspects of this study is that this is the first meta-analysis examining the efficacy of herbal treatments on HPV infection. The study aimed to provide less heterogeneous data by solely analyzing herbal treatments, contributing valuable insights to the field and literature. Another strength lies in the diverse participant background, with individuals from studies representing various income levels and different advantage-disadvantage groups, enhancing the generalizability of the findings.

The study's meticulous approach to scanning multiple databases and involving multiple researchers in the data extraction process ensures low bias and error. In addition, the methodological quality of the included studies was collaboratively assessed independently by each researcher, reaching a consensus after individual evaluations. However, one notable limitation is the inclusion of only English or Turkish publications, potentially excluding relevant studies published in other languages.

## CONCLUSION

According to the meta-analysis results, herbal treatments demonstrated a reduction in HPV infections. While herbal treatments were effective in decreasing atypical squamous cells of undetermined significance, no significant impact was observed in the regression of low-grade squamous intraepithelial lesions. Nevertheless, further meta-analyses considering the effectiveness of herbal treatments require more RCTs to draw a more conclusive result.
